# Cancer Transcriptome Dataset Analysis: Comparing Methods of Pathway and Gene Regulatory Network-Based Cluster Identification

**DOI:** 10.1089/omi.2016.0169

**Published:** 2017-04-01

**Authors:** Seungyoon Nam

**Affiliations:** ^1^ Department of Genome Medicine and Science, College of Medicine, Gachon University, Incheon, Korea.; ^2^ Department of Life Sciences, Gachon University, Seongnam, Korea.; ^3^ Gachon Institute of Genome Medicine and Science, Gachon University Gil Medical Center, Incheon, Korea.

**Keywords:** cancer transcriptomics, computational biology, gastric cancer, genomics, systems biology

## Abstract

Cancer transcriptome analysis is one of the leading areas of Big Data science, biomarker, and pharmaceutical discovery, not to forget personalized medicine. Yet, cancer transcriptomics and postgenomic medicine require innovation in bioinformatics as well as comparison of the performance of available algorithms. In this data analytics context, the value of network generation and algorithms has been widely underscored for addressing the salient questions in cancer pathogenesis. Analysis of cancer trancriptome often results in complicated networks where identification of network modularity remains critical, for example, in delineating the “druggable” molecular targets. Network clustering is useful, but depends on the network topology in and of itself. Notably, the performance of different network-generating tools for network cluster (NC) identification has been little investigated to date. Hence, using gastric cancer (GC) transcriptomic datasets, we compared two algorithms for generating pathway versus gene regulatory network-based NCs, showing that the pathway-based approach better agrees with a reference set of cancer-functional contexts. Finally, by applying pathway-based NC identification to GC transcriptome datasets, we describe cancer NCs that associate with candidate therapeutic targets and biomarkers in GC. These observations collectively inform future research on cancer transcriptomics, drug discovery, and rational development of new analysis tools for optimal harnessing of omics data.

## Introduction

Transcriptome analysis is broadly advocated for biomarker discovery, personalized medicine, and functional understanding of complex biological systems in health and disease states such as cancer (Karagoz et al., 2015; Mirsafian et al., 2016; Waldron and Riester, 2016). However, such analysis often faces considerable complexity, due to interdependencies between gene entries.

Networks from transcriptome datasets reveal regulatory relationships among biological entities, providing a systems scale understanding of molecular mechanisms (Barabasi and Oltvai, 2004). The importance of network generation has been widely accepted for addressing biological questions, including those in cancer pathogenesis (Aytes et al., 2014), and distinct algorithms have also been introduced to study networks. Broadly, there are two types of algorithms: signaling pathway networks (SPNs) and gene regulatory networks (GRNs). While many comparisons between GRN-generating algorithms have been performed, such comparisons between SPNs and GRNs, in terms of network clusters (NCs), have been little evaluated.

Network clustering provides clues for capturing important regions within complex network topologies, in terms of densely connected regions (i.e., “clusters”) (Morris et al., 2011). To obtain such clusters from high-throughput “omics” data, different frameworks can be used. While the SPN framework utilizes both prior signaling knowledge and publically available omics data, GRN uses only omics data, without prior information. Further transformation of networks (from these two frameworks) into clusters has not yet been attempted, in terms of which approach provides more informative NCs having cancer-related functional contexts.

Knowledge of intracellular oncogenic signal transduction reveals potential “druggable” molecular targets (Jia et al., 2009). Consequently, targeted cancer therapy requires *a priori* understanding of the molecular mechanisms involved in tumor pathogenesis (Barabasi and Oltvai, 2004; Berger and Iyengar, 2009; Jia et al., 2009). Since a network derived from high-throughput (“omics”) technologies involves a highly complex set of molecular mechanisms (Barabasi and Oltvai, 2004), it is not computationally feasible to incorporate all the signaling data from a particular network, for determining potential therapeutic strategies. Thus, these data must be “narrowed down” into subsets of molecular mechanisms, represented as NCs. As expected, this filtering process assumes that densely connected regions, or NCs, converge at functional “hubs” that may subsequently align with potential carcinogenic molecular mechanisms (Nam et al., 2012), for the potential discovery of effective “targeted” therapeutics (Barabasi and Oltvai, 2004; Berger and Iyengar, 2009; Goymer, 2008).

However, despite these promising approaches, it has yet to be demonstrated whether SPN or GRN methods yield more reliable NCs (in terms of cancer-functional contexts) (Morris et al., 2011). In this study, we compared our previously developed algorithm, PATHOME (*path*way and transcript*ome* information) (Nam et al., 2014), as an SPN method, with ARACNE (Algorithm for the Reconstruction of Accurate Cellular Networks) (Margolin et al., 2006), as a GRN method, in terms of agreement between NCs with a reference set (Futreal et al., 2004) of cancer-related functional contexts. The results of this comparison indicated that NCs of PATHOME, compared to those of ARACNE, better aligned with the reference set of cancer-functional contexts. We specifically used gastric cancer (GC), the fourth most worldwide common cancer type (Chang et al., 2016), as an example disease having few effective targeted therapies, due to limited understanding of its underlying biological bases (in terms of delineating network biology and clusters).

In sum, we applied PATHOME, and a network-clustering algorithm (Morris et al., 2011), to derive GC network-derived clusters (and potentially important therapeutic targets), in addition to improved mechanistic understanding of GC etiology. Also, the new observations reported in this study collectively inform future research on cancer transcriptomics, drug discovery, and rational development of new analysis tools for optimal harnessing of omics data.

## Materials and Methods

### Transcriptomic datasets

For comparing NCs, we obtained 3 GC RNA-Seq and microarray transcriptomic datasets, GEO (www.ncbi.nlm.nih.gov/geo) accessions GSE37023 (Wu et al., 2013), consisting of 112 GC tumors and 39 normal tissues; GSE36968 (Kim et al., 2012), containing 24 GC tumors and 6 noncancerous specimens; and GSE27342 (Cui et al., 2011), comprising 80 GC tumor samples and paired normal tissues (Table 1). These three datasets were used for constructing networks, as described below.

**Table 1.** Three Public Gastric Cancer Datasets in the Study

*GEO accession*	*Description*	*Experiment*	*References*
GSE37023	Gastric tumors vs. nonneoplastic gastric mucosa	Microarrays	Wu et al. (2013)
GSE36968	Gastric tumor samples vs. noncancerous gastric tissue samples	RNA-SEQ	Kim et al. (2012)
GSE27342	Paired tumor and adjacent nonneoplastic tissues from GC patients	Microarrays	Cui et al. (2011)

We used the three GC transcriptome datasets for comparing network cluster identifications and describing GC network clusters.

GC, gastric cancer.

### Construction of PATHOME and ARACNE networks

We first used “PATHOME” (Nam et al., 2014) to generate SPNs (henceforth, “PATHOME networks”) from analyzing the three transcriptomic datasets, using the default option (*p* < 0.05). Obtaining genes retained in the PATHOME network, for each dataset, we applied ARACNE (Margolin et al., 2006) to the matrix of expressed genes, to generate GRNs (henceforth, “ARACNE networks”) having different connections between the genes. In application of ARACNE, we used its default options.

### PATHOME-derived NCs versus ARACNE-derived NCs

For each dataset, NCs, from PATHOME and ARACNE networks, were identified using the Markov Clustering algorithm (MCL), implemented in clusterMaker (Morris et al., 2011). Using the NCs obtained by the two approaches, we performed two comparisons: first, whether the PATHOME or ARACNE networks contained more NCs; and second, we compared whether NCs derived from PATHOME networks (henceforth, “PATHOME-NCs”) or NCs derived from ARACNE networks (henceforth, “ARACNE-NCs”) contained more cancer-related functional contexts, represented matches to a set of Gene Ontology (GO) terms.

In the first comparison, since NCs represent functional “hubs” (i.e., convergence points for multiple pathways) (Barabasi and Oltvai, 2004; Berger and Iyengar, 2009; Goymer, 2008), the number of NCs included in any specific network is important. In the first comparison, we compared the number of PATHOME- and ARACNE-NCs (defined above), for all three transcriptome datasets. We further examined the obtained number of NCs, according to the total number of NC entries, using the two approaches.

For the second comparison, we set up a reference set of cancer-functional contexts associated with specific GO terms. By inputting the gene list (Futreal et al., 2004) of the cancer Gene Census repository (cancer.sanger.ac.uk/cosmic/census) into DAVID v6.7 (Huang et al., 2007) (under a significance cutoff as default EASE (Huang et al., 2007) score threshold of 0.1), we obtained 973 GO terms significantly associated with the gene list. The 973 GO terms (henceforth, reference GOs) were set as the reference set of cancer-related functional contexts, including various cancer “hallmark” (Hanahan and Weinberg, 2011).

To obtain significant reference GO terms associated with the PATHOME-NCs of a dataset, we only considered NCs having more than four genes. Collecting all the genes from such NCs, we fed the genes into DAVID (under a significance cutoff as default EASE (Huang et al., 2007), score threshold of 0.1), acquiring the number of significant GO terms. We used the default background, provided by DAVID, for the GO assessment.

We then assessed intersections between the significant GO terms of the PATHOME-NCs and the reference GOs, obtaining the number of cancer-functional contexts in PATHOME-NCs. The intersections correspond to the number of detected reference GOs. The same procedure was then repeated for obtaining and examining the number of cancer-functional contexts in ARACNE-NCs. Then, we compared the number of detected reference GOs between ARACNE-NCs and PATHOME-NCs, as reference cancer-related functional contexts.

## Results

### Topological difference between PATHOME- and ARACNE-derived networks

**FIG. 1.**
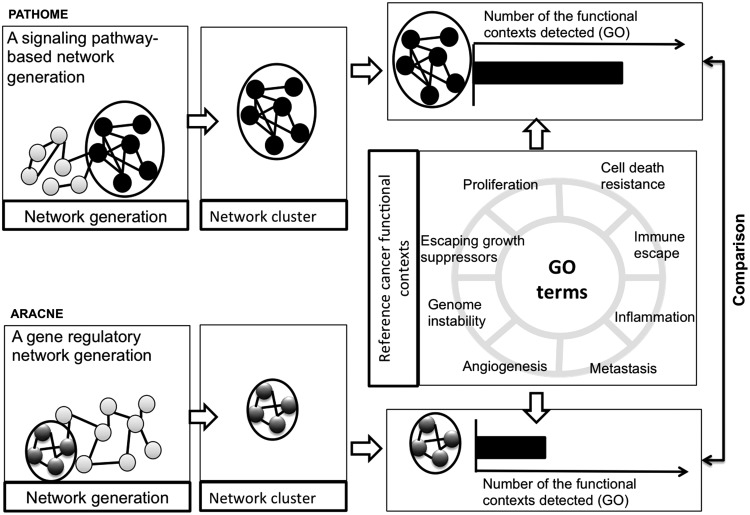
Overview of the study design and comparisons between PATHOME-NCs and ARACNE-NCs in analysis of three GC transcriptome datasets. Network clustering results depend on network generation methods. In this study, we compared the performance of two differently generated NCs, (PATHOME-NCs and ARACNE-NCs), using the algorithm MCL (Morris et al., 2011). Using GC datasets as examples, we measured whether PATHOME-NCs or ARACNE-NCs detected more cancer-related functional contexts, in terms of cancer-associated GO terms (equivalently, reference GOs). GC, gastric cancer; MCL, Markov Clustering algorithm; GO, Gene Ontology; NC, network cluster.

A schematic of our approach, using the PATHOME and ARACNE algorithms (Morris et al., 2011), is shown in Figure 1. Briefly, our objective was to compare how the two different network construction methods affected the identification of NCs (Fig. 1), based on each method's topologies. After network construction, we applied a Markov Clustering algorithm (MCL) (Morris et al., 2011) for identifying clusters (Fig. 1). We then inspected which method better reported a reference set of cancer-functional contexts (Fig. 1).

First, we characterized the network topological parameters [clustering coefficient, network centralization, network density, network diameter, network heterogeneity, and network radius; see descriptions in Supplementary Table S1 (Doncheva et al., 2012)] of the two methods, using the three above-mentioned datasets (Supplementary Fig. S1 and Supplementary Table S1) and the Cytoscape Network Analyzer plugin (Assenov et al., 2008). In terms of network diameter and heterogeneity, PATHOME (Nam et al., 2014) networks were larger than those of ARACNE (Margolin et al., 2006), for the three datasets (Supplementary Fig. S1 and Supplementary Table S2). With regard to network radii, ARACNE networks were larger than PATHOME networks, in the three datasets (Supplementary Fig. S1), while the clustering coefficients of PATHOME networks and ARACNE networks were nearly identical in two of the datasets [GSE27342 (Cui et al., 2011) and GSE36968 (Kim et al., 2012; Nam et al., 2014)], but differed for GSE37023 (Wu et al., 2013) (Supplementary Fig. S1).

Of additional interest, we observed that the GSE27342 and GSE36968 datasets showed similar patterns of all topological parameters, while GSE37023 (Wu et al., 2013) showed opposite patterns for clustering coefficient and network centralization (Supplementary Fig. S1), implying a different data context for the dataset GSE37023 compared to the other two datasets.

### Comparison between PATHOME- and ARACNE-derived NCs

We next characterized the PATHOME- and ARACNE-NCs. Since topologically densely connected regions are often regarded biologically critical cascades (Goymer, 2008; Morris et al., 2011), strongly associated with modes of disease mechanisms, acquisition of more NCs from a network is a meaningful comparison for the identification of candidate therapeutic targets (Berger and Iyengar, 2009). In our study, densely connected regions (in networks) refer to NCs identified by the network-clustering algorithm, MCL (Morris et al., 2011). Consequently, using MCL (Morris et al., 2011), we extracted densely connected regions from networks, obtaining more PATHOME-NCs than ARACNE-NCs, from the three real biological datasets: specifically, 81 versus 3, for GSE36968 (Kim et al., 2012; Nam et al., 2014); 81 versus 46, for GSE27342 (Cui et al., 2011); and 88 versus 57, for GSE37023 (Wu et al., 2013) for PATHOME- and ARACNE-NCs, respectively (Supplementary Table S3).

**FIG. 2.**
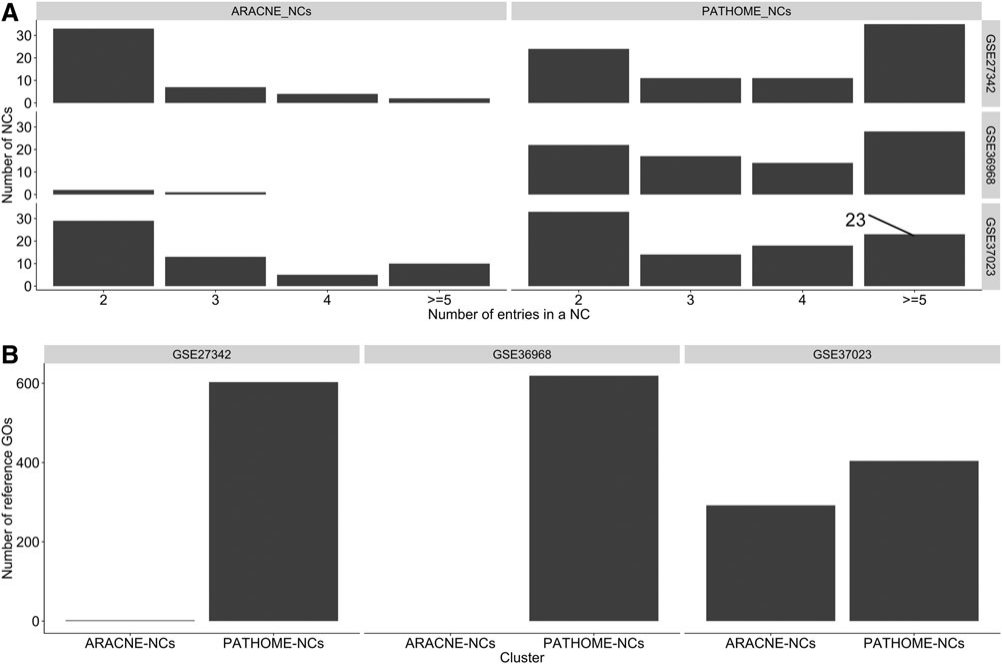
Comparisons between PATHOME-NCs and ARACNE-NCs in three GC transcriptome datasets. **(A)** *x*-axis represents the number of entries in specific NCs, while the *y*-axis represents the number of NCs. For example, in the GSE37023 dataset, the number of PATHOME-NCs, having five or more gene entries, was 23 (indicated by the *black line*). As a whole, PATHOME-NCs had more NCs having cluster entries ≥5, compared to ARACNE-NCs, as derived from the three datasets. These results indicate that PATHOME networks were fragmented into larger gene entry sizes of NCs, compared to ARACNE networks, when the same MCL was applied. **(B)** In the three GC datasets, the graph represents whether PATHOME-NCs or ARACNE-NCs detected (or contained) more reference cancer-functional GO terms (*y*-axis). For example, in GSE37023, PATHOME-NCs detected 404 reference cancer-functional GO terms, and ARACNE-NCs detected 292 reference GO terms.

Therefore, PATHOME-NCs may reveal higher levels of pathway signaling associated with potential therapeutic targeting options, when compared to ARACNE. Also, further dissecting the number of NCs, according to the number of NC entries, PATHOME-NCs reported more NCs with entries (or nodes) greater than or equal to 5, when compared to ARACNE-NCs (Fig. 2A). This indicates that PATHOME-NCs report NCs in a collective, but not fragmented, manner.

### Comparing PATHOME- and ARACNE-derived cancer-associated NCs

In addition to the previously described analyses, to identify whether PATHOME- or ARACNE-NCs detected more cancer-related functional contexts, we set reference GO terms significantly associated with the cancer Gene Census (Futreal et al., 2004) as the reference set of cancer-functional contexts (reference GOs; see Materials and Methods section in detail).

In the dataset GSE27342, PATHOME-NCs reported 1098 statistically significant GO terms, with 603 (Fig. 2B) overlapping with cancer reference GOs. In the same dataset, ARACNE-NCs reported five significant GO terms, two (Fig. 2B) of which matched the reference GOs. In dataset GSE37023, by contrast, PATHOME-NCs reported 612 significant GO terms, 404 of which overlapped reference GOs, while ARACNE-NCs reported 451 significant GO terms, with 292 overlapping the reference GOs (Fig. 2B). In dataset GSE36968, ARACNE-NCs, having at least five nodes, were not found, resulting in no GO terms; whereas PATHOME-NCs having at least five nodes reported 1063 significant GO terms, with 620 of these overlapping the reference GOs (Fig. 2B). Thus, PATHOME-NCs better matched the cancer-functional contexts, compared to ARACNE-NCs, throughout the comparisons, using the three independent datasets. We confirmed that PATHOME network clustering showed better cancer network identification performance compared to ARACNE. We describe several PATHOME-NCs from the three datasets in the Discussion section.

## Discussion

There is a growing need for innovation in data analysis algorithms and bioinformatics in the context of cancer transcriptomics. As shown in the Supplementary Figure S1, topology parameter differences between PATHOME and ARACNE networks indicate different connectivity patterns. In particular, network diameters (the longest distance between any two nodes) of PATHOME network were consistently longer than those of ARACNE network. This may partly be due to PATHOME's consideration of paths consisting of consecutive edges of more than two nodes (Nam et al., 2014), while ARACNE only considers single edges (without regard to consecutive single edges).

**FIG. 3.**
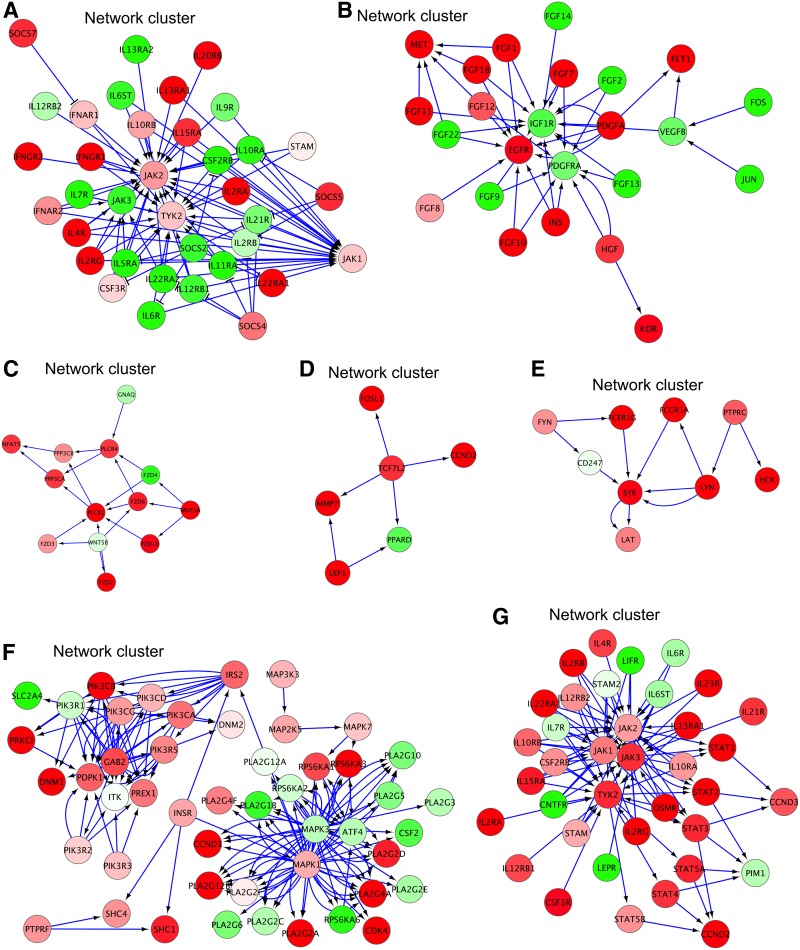
Several selected NCs from the GC datasets by PATHOME network-derived network clustering method. NCs A–D were derived from GSE36968, and NCs E–G were obtained from GSE27342. The *red* node colors indicate upregulated genes in GC tumors (compared to normal tissue), while the *green* node colors represent downregulated genes.

GC, with a worldwide incidence of 952,000 cases and 723,000 deaths (Torre et al., 2015), currently has few nonsurgical therapeutic options (Bang et al., 2010). Toward improving these dire statistics, acquisition of GC-related NCs (as we performed in this study) could facilitate the identification of druggable signaling cascades/networks (Fig. 3).

The NC A was the largest NC ascertained from GSE36968 (transcriptome dataset of Asian GC tumors vs. noncancerous tissues), implicating multiple STAT proteins and JAK family kinases related to immune response and hematopoiesis (Ubel et al., 2013). In this specific cluster, the JAK family kinase genes *TYK2* and *JAK2* (among others) were upregulated in GC, compared to normal, tissues. Upregulation of JAK members has been well reported in breast, prostate, and cervical cancers, playing diverse roles in differentiation and cancer cell proliferation and survival (Rane and Reddy, 2000). Moreover, it was recently reported that JAK/STAT pathways upregulate programmed death receptor ligand 1 (PD-L1) in cancer cells, an event that leads to evasion of T cell-mediated antitumor immune responses (Ohaegbulam et al., 2015; Ritprajak and Azuma, 2015). Thus, this cluster suggests the feasibility of immunotherapy in GC as a new therapeutic option, considering that GC has very limited effective targeted therapies (Bang et al., 2010).

In addition to NC A, NC B (as derived from GSE36968; Fig. 3) showed upregulation of the proto-oncogenes *KDR*, *FLT1*, *EGFR*, and *MET* in GC tumors, compared to normal gastric tissues. Of these, KDR and FLT1 are both receptors for VEGFA, the predominant mediator of angiogenesis in tumor progression (Ferrara and Adamis, 2016; Slattery et al., 2014). Thus, this finding links GC to VEGF signaling, a common therapeutic target, in numerous other advanced cancer types, over the last decade (Ferrara and Adamis, 2016; Goel and Mercurio, 2013).

Moreover, tumor-synthesized VEGF is an important immunosuppressive cytokine for eluding immunosurveillant cells (e.g., NK cells, T cells, and so on) (Dunn et al., 2006; Matsueda and Graham, 2014; Nam and Park, 2012), and MET and EGFR are also strong therapeutic target candidates in numerous cancers, including those of the lung and colorectum (Cataisson et al., 2016; Gou et al., 2016; Misale et al., 2014; Pietrantonio et al., 2016; Shida et al., 2004; Takahashi et al., 2016). In metastatic colorectal cancer, amplification and overexpression of *MET* is a key contributing factor to resistance to anti-EGFR therapies (Misale et al., 2014; Takahashi et al., 2016). Furthermore, dual inhibition of MET and EGFR by “biseptic” antibodies, or combined inhibitors, demonstrated effective inhibition of tumor growth *in vitro* and *in vivo* (Castoldi et al., 2013; Lee et al., 2016a; Xu et al., 2011), in accord with our findings in GC cluster B. Recently, another dual inhibition of MET and EGFR in GC cells has emerged, based on inhibition of sphingosine 1-phosphate (S1P), a G protein-coupled receptor ligand (Shida et al., 2004).

Clusters C and D (Fig. 3), from the GSE36968 transcriptome dataset, contained both upregulated upstream and downstream WNT pathway effector genes, including *WNT5A*, *TCF7L2*, and *LEF1*, in accord with our previous studies (Chang et al., 2016; Nam et al., 2014). In addition, it has been found that WNT5A is an emerging druggable GC signal mediator, through transcriptional regulation by HNF4α (Chang et al., 2016; Nam et al., 2014), while another study (Chang et al., 2016) indicated poor prognosis of WNT5A high-expressing patients, in particular, in Lauren-classified diffuse-type GC patients. That study (Chang et al., 2016) further demonstrated WNT5A-mediated signal inhibition, through HNF4α antagonism by various “rationally designed” small compounds. That finding agrees with our results in this study, showing clusters C and D to associate with druggable GC signaling, as well as showing promising utility as biomarkers.

MCL analysis of PATHOME-delineated networks from GC tumor versus paired adjacent normal tissue transcriptomes (GEO: GSE27342) resulted in the identification of 35 NCs. The largest cluster (cluster F in Fig. 3) showed upregulation of multiple kinase genes (Fleuren et al., 2016), including *PRKCI*, *INSR*, *RPS6KA1*, *CDK4*, and *MAPK1* (encoding the mitogen ERK2). These five gene products associate with the receptor tyrosine kinase pathways PI3K-mTOR and MAPK, mediating the cell cycle and numerous core cellular processes and pathways (Fleuren et al., 2016).

Moreover, since “omics” profiling of kinases revealed context-specific functions, according to cancer tissue types (Fleuren et al., 2016), cluster F may associate with specific GC tumor-oriented kinome characteristics. Also, inhibitors of kinases found upregulated in this cluster could be repurposed, from other cancers, to GC. For example, the signal transducers, RPS6KA1 and MAPK1, found in cluster F, also associate with other cancer tissue types (Kyriakis and Avruch, 2001; Lara et al., 2011; Salhi et al., 2015; Zhao et al., 1995). Of these, ERK2 (encoded by the gene *MAPK1*) was reported to increase the activity and phosphorylation of the oncoprotein RPS6KA1 (ribosomal protein S6 kinase, 90 kDa, polypeptide A1), leading to cancer cell proliferation (Kyriakis and Avruch, 2001; Zhao et al., 1995). RPS6KA1 has also been recognized as a therapeutic target in lung cancer (Lara et al., 2011) and nodular type melanoma (Salhi et al., 2015).

NCs E and G (Fig. 3), derived from the GSE27342 dataset (GC tumors vs. adjacent normal tissues), revealed involvement of the mitogenic Syk/Lyn and JAK/STAT pathways, respectively. Specifically, the spleen tyrosine kinase (SYK, in cluster E), expressed in majority of hematopoietic cells, has been recognized as a therapeutic target in autoimmune diseases such as rheumatoid arthritis (Geahlen, 2014).

In addition, SYK has been recently recognized as a prosurvival factor in breast cancers, as well as hematopoietic malignancies (Lee et al., 2016b), representing a strong candidate for anticancer therapy (Geahlen, 2014). In fact, fostamatinib, cerdulatinib, and entospletinib, as Syk inhibitors, are under clinical investigation as single or adjuvant agents, in certain types of leukemia (Coffey et al., 2014; Sharman et al., 2015). LAT (Linker For Activation Of T-Cells), in this same cluster, is a downstream effector of Syk signaling, and *in vitro LAT* knockdown decreased proliferation, as well as migration, of GC cell lines (Wang et al., 2013).

Moreover, cluster E revealed a potentially druggable GC target, including possible “repurposing” of cerdulatinib, a dual inhibitor of Syk and JAK/STAT signaling in certain types of diffuse large B cell lymphoma cell lines (Ma et al., 2015). This suggests likely GC association with NC G, also consisting JAK/STAT signaling. Therefore, a synergistic effect, by downregulation of NCs E and G, should be established for developing new GC therapeutic strategies.

We also identified the consensus genes of the PATHOME-NCs among the three datasets. Restricting to PATHOME-NCs containing greater than or equal to five genes, we compared the genes for the PATHOME-NCs between the three datasets. As a result, we found 153 consensus genes (Supplementary Fig. S2). Out of the 153 genes (Supplementary Table S4), *EGFR*, *JAK2*, *JAK3*, *MAPK1*, *TYK2*, and *WNT5A* resided in the aforementioned NCs. Again, those genes associated with JAK/STAT as well as WNT signaling.

## Conclusions

The use of PATHOME-NCs showed better relevance to cancer, in agreement with a reference set of cancer-functional contexts, compared to ARACNE-NCs. The JAK/STAT signaling, in GC PATHOME-NCs, has now been revealed in the pathogenesis of many diverse cancer types, holding promise for the application of effective targeted GC therapies or personalized medicine in this devastating type of cancer. Furthermore, rigorous analytical comparisons (Abu-Asab et al., 2008) and experimental validation are necessary to advance this line of research on cancer transcriptome data analysis.

## Supplementary Material

Supplemental data

Supplemental data

Supplemental data

Supplemental data

Supplemental data

Supplemental data

## Abbreviations Used

ARACNE: Algorithm for the Reconstruction of Accurate Cellular Networks
GC: gastric cancer
GO: Gene Ontology
GRNs: gene regulatory networks
LAT: Linker For Activation Of T Cells
MCL: Markov Clustering algorithm
NC: network cluster
PATHOME: *path*way and transcript*ome* information
SPNs: signaling pathway networks
SYK: spleen tyrosine kinase
