# Supplementary material for: Cancer Transcriptome Dataset Analysis: Comparing Methods of Pathway and Gene Regulatory Network-Based Cluster Identification
Source: OMICS. 2017 Apr 1;21(4):217–24. doi: 10.1089/omi.2016.0169 (PMC5393410; doi:10.1089/omi.2016.0169)
Supplement: Supplemental data [file Supp_Table1.pdf]

Supplementary Table S1. Definition of the Network Parameters

Network parameters	*Description*
Clustering coefficient	The clustering coefficient of a node represents the modularity of a specific network (Doncheva et al., 2012) and is proportional to the number of neighbors over the maximum path length (reaching to the node's neighbors). We obtained the average clustering coefficients of all the nodes, reporting the value in Supplementary Figure S1.
Network centralization	Network centralization (ranging from 0 to 1) measures how close a network's topology is to star-like (equivalently, centralized). Zero indicates that networks are uniformly (equivalently, decentralized) connected networks.
Network density	Network density is defined as the average number of neighbors of nodes. Also the values range from 0 to 1.
Network diameter	The parameter is defined as the longest distance between any two nodes (Doncheva et al., 2012).
Network heterogeneity	The parameter indicates the tendency to likely have hub nodes in network (Doncheva et al., 2012).
Network radius	The parameter is the nonzero shortest (Doncheva et al., 2012) path length between two nodes. If a node is isolated, the value is close to 1.
