# Supplementary material for: Cancer Transcriptome Dataset Analysis: Comparing Methods of Pathway and Gene Regulatory Network-Based Cluster Identification
Source: OMICS. 2017 Apr 1;21(4):217–24. doi: 10.1089/omi.2016.0169 (PMC5393410; doi:10.1089/omi.2016.0169)
Supplement: Supplemental data [file Supp_Fig1.pdf]

Supplementary Materials

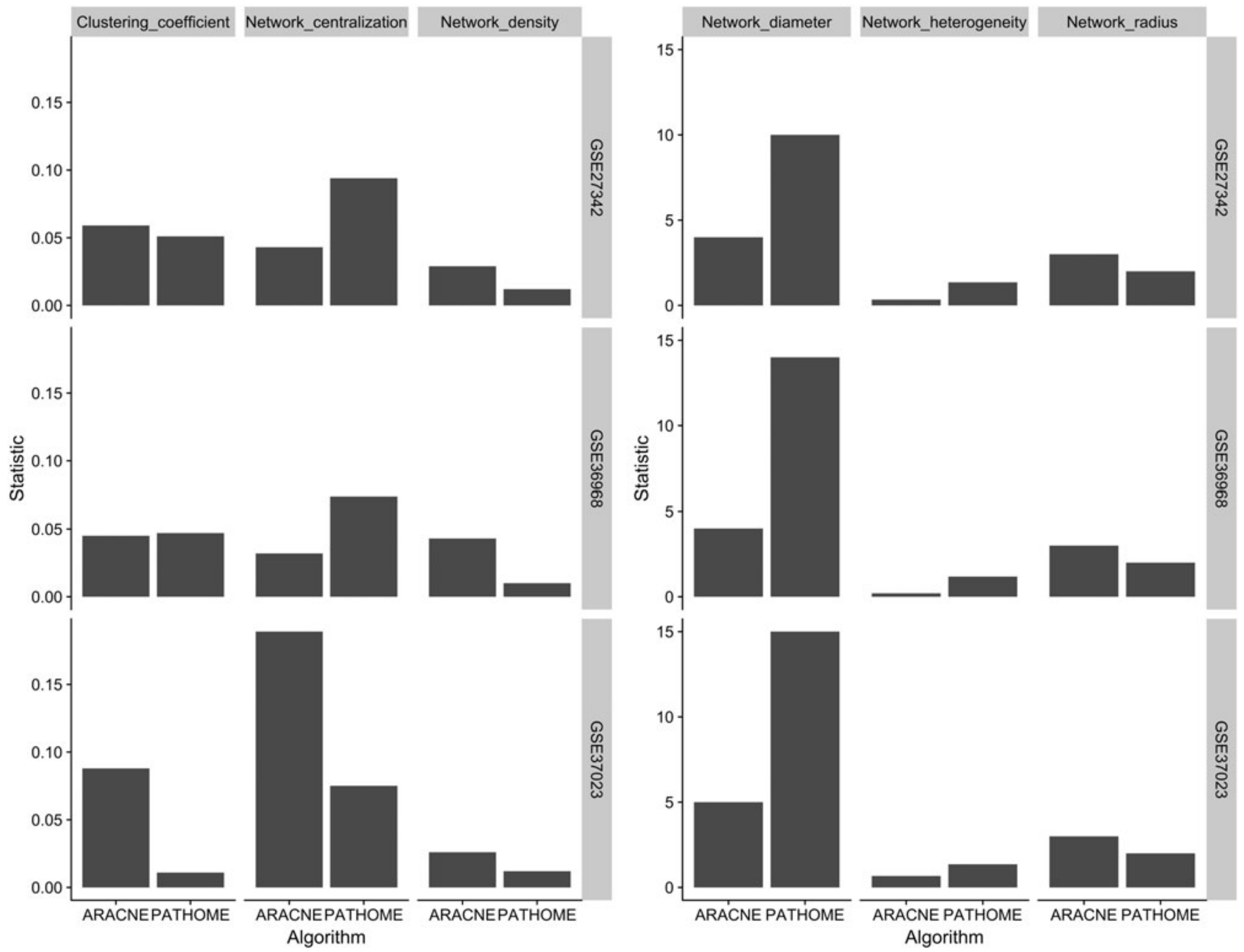

SUPPLEMENTARY FIG. S1. Topology parameters of PATHOME networks and ARACNE networks in three GC datasets (GSE27342, GSE36968, and GSE37023). The six topology parameters (clustering coefficient, network centralization, network density, network diameter, network heterogeneity, and network radius), as detected by clusterMaker, are represented. GC, gastric cancer; NC, network cluster.
