# Supplementary material for: Cancer Transcriptome Dataset Analysis: Comparing Methods of Pathway and Gene Regulatory Network-Based Cluster Identification
Source: OMICS. 2017 Apr 1;21(4):217–24. doi: 10.1089/omi.2016.0169 (PMC5393410; doi:10.1089/omi.2016.0169)
Supplement: Supplemental data [file Supp_Table2.pdf]

Supplementary Table S2. Topology Parameters of PATHOME- and ARACNE-Derived Networks

| *Dataset* | *GSE27342* | | *GSE36968* | | *GSE37023* | |
|---|---|---|---|---|---|---|
| *Items/algorithms* | *PATHOME* | *ARACNE* | *PATHOME* | *ARACNE* | *PATHOME* | *ARACNE* |
| Clustering coefficient | 0.051 | 0.059 | 0.047 | 0.045 | 0.011 | 0.088 |
| Network centralization | 0.094 | 0.043 | 0.074 | 0.032 | 0.075 | 0.189 |
| Network density | 0.012 | 0.029 | 0.01 | 0.043 | 0.012 | 0.026 |
| Network diameter | 10 | 4 | 14 | 4 | 15 | 5 |
| Network heterogeneity | 1.354 | 0.341 | 1.179 | 0.203 | 1.36 | 0.675 |
| Network radius | 2 | 3 | 2 | 3 | 2 | 3 |

The table is equivalent to Supplementary Figure S1.
