# Supplementary material for: Cancer Transcriptome Dataset Analysis: Comparing Methods of Pathway and Gene Regulatory Network-Based Cluster Identification
Source: OMICS. 2017 Apr 1;21(4):217–24. doi: 10.1089/omi.2016.0169 (PMC5393410; doi:10.1089/omi.2016.0169)
Supplement: Supplemental data [file Supp_Table3.pdf]

Supplementary Table S3. Comparisons Between PATHOME-NCs and ARACNE-NCs in Three GC Transcriptome Datasets

Dataset	*GSE27342*		*GSE36968*		*GSE37023*	
The number of NC entries	*ARACNE-NC*	*PATHOME-NC*	*ARACNE-NC*	*PATHOME-NC*	*ARACNE-NC*	*PATHOME-NC*
≥5	2	35	0	28	10	***23***
4	4	11	0	14	5	18
3	7	11	1	17	13	14
2	33	24	2	22	29	33
Sum	46	81	3	81	57	88

The table is equivalent to Figure 2A. For example, in the GSE37023 dataset, the number of PATHOME-NCs, having five or more gene entries, was 23 (indicated in italics and bold).

GC, gastric cancer; NC, network cluster.
