# Supplementary material for: Cancer Transcriptome Dataset Analysis: Comparing Methods of Pathway and Gene Regulatory Network-Based Cluster Identification
Source: OMICS. 2017 Apr 1;21(4):217–24. doi: 10.1089/omi.2016.0169 (PMC5393410; doi:10.1089/omi.2016.0169)
Supplement: Supplemental data [file Supp_Fig2.pdf]

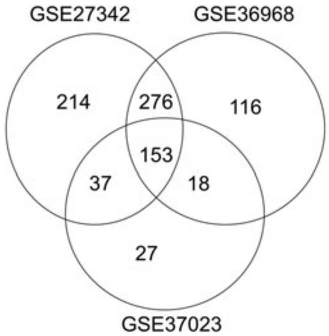

SUPPLEMENTARY FIG. S2. Consensus genes of the PATHOME-NCs between the three datasets. We inspected the gene entries of the PATHOME-NCs from the three datasets (GSE27342, GSE36968, and GSE37023). As a result, 153 genes were found to overlap between the three datasets.
