# Supplementary material for: Cancer Transcriptome Dataset Analysis: Comparing Methods of Pathway and Gene Regulatory Network-Based Cluster Identification
Source: OMICS. 2017 Apr 1;21(4):217–24. doi: 10.1089/omi.2016.0169 (PMC5393410; doi:10.1089/omi.2016.0169)
Supplement: Supplemental data [file Supp_Table4.pdf]

Supplementary Table S4. The 153 Consensus Genes in Supplementary Figure S2

| | | | | | | | | | | | | | |
|---|---|---|---|---|---|---|---|---|---|---|---|---|---|
| HRAS | MAPK3 | HGF | GNG11 | EGFR | ARPC3 | CACNA1C | IL6ST | KRAS | CXCL3 | FYN | IRAK4 | CXCR4 | TNF |
| OSMR | GNAI3 | FGF7 | IL7R | RELA | ARPC2 | MAX | STAT5B | LAMB1 | CXCL2 | PDGFB | ELK4 | SHC1 | XIAP |
| CCR10 | GNAI2 | IL4R | PTK2B | ARPC1A | TRAF6 | NCOA4 | STAM | NTRK3 | IL10RA | PDGFA | ITGB8 | CSK | PIK3R5 |
| ILK | GNAI1 | IL13RA1 | PLA2G12A | ARPC1B | TRAF4 | ARPC5L | BCR | LAMA2 | PLA2G10 | CSF3R | ITGAV | IFNGR2 | IL2RG |
| PIK3CA | NFKBIB | LYN | PPP3CA | TRAF1 | TRAF3 | SH2B3 | CCR7 | CBLB | FZD3 | PDGFC | ITK | AKT3 | PIK3R3 |
| GNG4 | ERBB2 | IL6R | PDK1 | CCL2 | VAV1 | NFATC4 | LAMC3 | LAMA4 | BIRC3 | FGFR2 | ITGA2 | AKT2 | PIK3R1 |
| GNG5 | NFKBIA | CACNG2 | NRAS | FGR | ATF4 | COL4A2 | PDGFRB | LAMA3 | BIRC2 | WNT5A | ITGA3 | PRKCA | PIK3R2 |
| PIK3CG | BCL2L1 | CCND1 | ARAF | ARPC5 | CACNA1H | COL4A1 | LAMC2 | NTRK2 | STAT3 | ITGB4 | ITGA5 | IFNAR1 | ACTB |
| PIK3CB | RAC1 | GNB2 | IQGAP2 | MAPKAPK2 | CACNA1E | JAK1 | LAMC1 | WASF2 | FZD7 | ITGB5 | ITGA7 | PRKCB | IL2RB |
| PIK3CD | MAP2K1 | GNB1 | IL15RA | IL12RB2 | ABL1 | JAK2 | PXN | NFKB1 | STAT2 | ITGB1 | MTOR | TYK2 | CRK |
| MAPK1 | MAP2K2 | CSF2 | RRAS | DOCK1 | IKBKB | JAK3 | LAMB3 | PLA2G6 | MAPK13 | SRC | PLCB3 | CRKL | |

The 153 consensus genes of the PATHOME-NCs between the three datasets.
